# Autophagy is associated with cell fate in the process of macrophage-derived foam cells formation and progress

**DOI:** 10.1186/s12929-016-0274-z

**Published:** 2016-07-30

**Authors:** Xiaopeng Liu, Yue Tang, Yongchun Cui, Hong Zhang, Dong Zhang

**Affiliations:** Animal experimental center & Beijing Key Laboratory of Pre-clinical Research and Evaluation for Cardiovascular Implant Materials, State Key Laboratory of Cardiovascular Disease, National Center for Cardiovascular Diseases, Fu Wai Hospital, Chinese Academy of Medical Sciences and Peking Union Medical College, Beijing, 100037 People’s Republic of China

**Keywords:** Autophagy, Macrophages, Foam cells, ApoE−/− mice, Atherosclerosis

## Abstract

**Background:**

Autophagy participates in plaque formation and progression; however, its association with foam cells’ fate is unknown. To investigate autophagy features and its effect on the fate of different-stage macrophage foam cells (FCs). Different-stage FCs were obtained through incubation of THP-1 macrophages (THP-M) with oxidized low-density lipoprotein LDL (oxLDL, 80 μg/mL) for various durations (0–72 h). Autophagy in THP-1 macrophage FCs and in apoE−/− mice was regulated by Rapamycin (80 ug/mL) or 3-MA (10 mM). Lipid droplet accumulation, LC3 I/II, P62 expression level, and autophagic flux were measured. Vascular ultrasound, TUNEL, IHC, and DHE staining were used to detect the artery plaques in apoE−/− mice.

**Results:**

In early-stage FCs, the amount of autophagosomes gradually increased, and autophagic flux intensity accelerated, but in mid-late stage FCs, autophagic flux was suppressed. For early stage FCs, treatment with autophagy activator rapamycin markedly decreased intracellular lipid content and prevented them from transforming into foam cells, while the autophagy inhibitor 3-MA considerably increased the intracellular lipid-droplet accumulation. During the process of foam cell development, upregulating autophagy not only reduced intracellular lipid-droplet accumulation, but also inhibited cell apoptosis through clearing dysfunctional mitochondria and lowering intracellular ROS level. The in vivo experiments produced consistent results that rapamycin administration in apoE−/− mice reduced the death rate of macrophages and delayed plaque progression.

**Conclusions:**

The fate of macrophage FCs was associated with autophagy. Early autophagy enhancement inhibits the formation and progression of macrophage FCs and prevents atherosclerosis.

**Electronic supplementary material:**

The online version of this article (doi:10.1186/s12929-016-0274-z) contains supplementary material, which is available to authorized users.

## Background

Autophagy is an evolutionarily conserved mechanism and the only physiological self-protective process through which organisms remove damaged or unwanted intracellular materials, including protein aggregates, lipid droplets, dysfunctional mitochondria, and other damaged intracellular organelles. The autophagic process is classically considered to be a pathway contributing to cellular homeostasis and adaptation to stress [1, 2]. Under the action of stress factors, basal autophagy may act as a pro-survival mechanism in an adverse environment [3, 4]. However, dysfunctional autophagy is associated with human disease. Defects in autophagy have been implicated in the pathogenesis of cancers, heart failure, cancer, neurodegeneration [5], and inflammatory diseases [6–8]. However, little is known about the role of autophagy in atherosclerosis.

A limited number of clinical studies have shown that autophagic markers are co-localized with macrophages in atherosclerotic plaques, and autophagy is impaired in the advanced stages of atherosclerosis; its deficiency induced lethal accumulation of cholesterol crystals and promoted atherosclerosis [9–11]. Stent-based delivery of the mTOR inhibitor everolimus promotes a stable plaque phenotype. However, the direct evidence indicating the role of autophagy is insufficient. Monocytes/macrophages are the predominant cell type expressing autophagy markers in the plaque [12]. Several basic examinations also revealed that mice with a macrophage-specific deletion of the essential autophagy gene *Atg5* developed plaques with increased apoptosis and oxidative stress and exhibited enhanced plaque necrosis [13], suggesting that autophagy is involved in AS pathology. Nevertheless, little is known about the regulation and mechanism associated with autophagy in the pathogenesis of atherosclerosis [10, 14, 15]. There are still some important questions to be elucidated, including changes in autophagy with AS progression, critical time points for correcting dysfunctional autophagy, and the effective regulation of autophagy to achieve a positive effect in inhibiting atheroma progression.

The present study was designed to address these issues using oxidative low-density lipoproteins (ox-LDL)-treated THP-1 macrophages and high-fat–fed Apo E −/− mice. We investigated the characteristics of autophagy at different stages of the development of THP-1 macrophage (THP-M)-derived foam cells and explored its mechanism of action and effect on middle-late foam cell viability. Mechanistically, this process, in part, involves mitochondrial oxidative stress and cell apoptosis. In Apo E −/− mice, the suitable upregulation of autophagy delays the progress of atherosclerotic plaques.

## Methods

### Culture and differentiation of THP-1-derived macrophages

Ox-LDL-treated THP-1 macrophage is a commonly used model in the studies on autophagy associated with atherosclerosis. Initially, THP-1 cell (ATCC, Manassas, VA, USA) was cultured in RPMI-1640 medium (Invitrogen, San Diego, CA, USA) supplemented with 20 U/mL penicillin (Invitrogen), 20 μg/mL streptomycin (Invitrogen), and 10 % fetal bovine serum (FBS) (Lonza, Walkersville, MD, USA). All cells were cultured at 37 °C in a 5 % CO_2_ environment, and the cellular medium was changed every 2–3 days. Cells were passaged upon reaching 80 % confluence, and all experiments were performed using cells at passage eight or lower. Then, to induce FC differentiation, THP-1 cells were incubated with 10^−7^ M phorbol 12-myristate 13-acetate (PMA) (Sigma-Aldrich) for 48 h, followed by incubation with 80 μg/mL oxLDL (Intracel Resources, Frederick, MD, USA) for 0, 6, 24, 48, and 72 h to form foam cells at differential stages.

### Oil red staining

To identify the lipid acumination at different stages of foam cell formation, after incubation with oxLDL for 0, 6, 24, 48, or 72 h, THP-M were stained with Oil Red (Sigma-Aldrich, MO, USA) for 10 min at RT. The Oil Red staining allowed for visualization and imaging of FC containing intracellular lipid droplets *via* a Leika microscope (Nikon Inc., Melville, NY, USA) at an objective magnification of 20×. The cells were photographed with a Coolsnap ES camera (Photometrics, Tucson, AZ, USA), using Simple PCI image capture software (Hamamatsu Corporation, Sewickley, PA, USA).

### MTT assay

Cell viability was measured by the MTT assay (M5655, Sigma-Aldrich, Inc., Saint Louis, MO, USA), based on the MTT conversion into formazan crystals *via* the action of mitochondrial dehydrogenases. Briefly, THP-M-derived foam cells were plated at a density of 2.5 × 10^4^ cells/cm^2^ in 96-well plates. After the treatment, the culture medium was replaced with 200 μL of MTT solution (5 mg/mL stock solution in PBS, diluted with culture medium to the final concentration 0.5 mg/mL). After 4-h incubation at 37 °C, this solution was removed, and the produced formazan was solubilized in 150 μL dimethyl sulfoxide (DMSO). The absorbance was measured at 570 nm through an automated microplate reader (Tecan Infinite 200 pro microplate reader, Männedorf, Switzerland). Cell viability was calculated by comparing the results to those of the control cells, which were considered 100 % viable.

### Flow cytometry

Detection of apoptosis and mitochondrial superoxide production was performed as previously described (17). Samples were analyzed using a BD FACSCanto II flow cytometer (BD Biosciences, CA, USA). For Annexin V-FITC/PI staining, the maximum FITC excitation wavelength/emission wavelength was 488 nm/525 nm, and the maximum PI excitation wavelength/emission wavelength was 535 nm/615 nm, respectively. A number of 10,000 events were collected for each sample. The CellQuest software (Becton Dickinson, San Jose, CA, USA) was used to analyze the data obtained.

### mRFP-GFP-LC3 plasmid construction and transfection

Microtubule-associated protein light chain 3 (LC3) is an ubiquitin-like protein that binds to autophagosomes (AVs). Typically, mammalian cells are transfected with GFP-tagged LC3 to track and follow the fate of AVs in the cell and to measure the autophagic flux. Here, we used an adenovirus (Ad-LC3-GFP-mRFP) expressing the GFP and mRFP fluorescent proteins for marking and tracking LC3 (HANBIO, Shanghai, China). In brief, 1640 medium (containing 10 % FBS) was employed to culture THP-1 cells which were incubated with the Ad-LC3-GFP-mRFP adenovirus for two hours to be infected. Then, the culture medium was replaced and the incubation continued for 36 h. Counting of autophagosomes and autolysosomes was performed on a Thermo Scientific Cellomics ArrayScan HCS reader (Thermo Fisher Scientific, Pittsburgh, PA, USA) (20x objective) using the Spot detector V3 Cellomics Bioapplication (Cellomics, USA.) GFP and RFP vesicles were identified on separate secondary channels by FITC- and Texas Red-associated filters, respectively, depending upon size, shape, and intensity thresholding. The mean number of vesicles per cell (object) was calculated by the ArrayScan software, as the total number of vesicles per field was divided by the total number of objects per field. One thousand cells were counted per coverslip, and the analysis was conducted on triplicate samples at least twice. The yellow spots and red spots after overlapping represented autophagosomes and autolysosomes, respectively. Through counting the different color spots, we determined the presence of autophagy in the different groups.

### Measurement of mitochondria oxidative stress

Mitochondria-derived superoxide anion in THP-M cells of different stages was determined quantitatively by an ELISA method based on Mito SOX (Molecular Probes), according to the manufacturer’s protocol. Superoxide anion generation was also established semi-quantitatively by fluorescence intensity as detected by the Image-Pro Plus software in conjunction with a Leica Confocal microscope.

### Western blotting

Aliquots of 20 mg of protein extracts from human THP-1 macrophages were separated by 10 % sodium dodecyl sulfate-polyacrylamide gel electrophoresis (SDS-PAGE) and subjected to immunoblotting with the following antibodies LC3B (Santa Cruz Biotechnology, Santa Cruz, CA, USA) and P62, GAPDH (1:1000) [16–19]. The densities of the bands were measured using a Densitograph System (Ez-Capture II and CS Analyzer 3.0, ATTO, Tokyo, Japan), and the ratio to GAPDH was calculated.

### Animal experiments

The animal experiments were approved by the Institutional Animal Care and Use Committee of Fuwai Hospital (Permission No. 2013-6-40-GZR). A total of 24 8-week-old male spontaneously hyperlipidemic ApoE−/− mice were purchased from the Institute of Laboratory Animal Sciences, Chinese Academy of Medical Sciences (CAMS) & Peking Union Medical College (PUMC) and fed with a high-fat rodent diet containing 21 % fat from lard and supplemented with 0.15 % (wt/wt) cholesterol (Special Diets Services) until 16 weeks of age (18). At that age, 24 mice were allocated by body weight and plasma total cholesterol levels into three groups (10 animals/group): the vehicle group, the Rapa group, and the 3-MA group. The animals were administered either the vehicle or the respective drug by i.p. route once a day for 6 weeks.

### Intravascular ultrasonic imaging

Mice were anesthetized using 10 % chloral hydrate (3 mL/100 g). Echocardiography was performed with a Vevo2100 ultrasound system (VisualSonics Inc., Toronto, Canada) and a MS400 transducer. Intravascular ultrasonic imaging was conducted to compare the effect of autophagy on plaque formation. Echocardiograms were interpreted by two separate cardiologists who were blinded to the animal groups.

### Histologic examination of atherosclerosis

At 24 weeks of age, ApoE^−/−^ mice were anesthetized with diethyl ether. The aortic root was carefully excised and fixed with 4 % formaldehyde and 10 % sucrose. The sections were stained with hematoxylin-eosin (HE) and oil red O to measure the total lesion area. The degree of intimal thickening was evaluated as previously described (14). Briefly, the lesion area of the intima and the luminal circumference of the media in the oil red-stained section were measured by an image analyzer. The intimal thickness (mm) was then calculated by dividing the intimal lesion area by the luminal circumference of the media. The cellular components of the atherosclerotic lesion in the thoracic aorta lesion were examined immunohistochemically by the streptoavidin method (LSAB Kit, Dako Japan Co, Ltd, Tokyo, Japan) using an anti-macrophage antibody (MOMA-2) as the primary antibody and a biotin-labeled anti-mouse IgG antibody and a biotin-labeled anti-sheep IgG antibody (MBL Co, Ltd, Nagoya, Japan) as the secondary antibodies, respectively. The following parameters were measured: plaque cross-sectional area, media cross-sectional area, percentage of occlusion of the lumen by plaque, the number of buried fibrous caps, current fibrous cap thickness, and percentage of lipid content of the plaque. An assessment of the oxidative stress in aortic root frozen-tissue sections was performed by fluorescence microscopy of dihydroethidium (DHE)-stained sections as previously described [16].

### Statistical analysis

Statistical significance was calculated *via* the unpaired Student’s *t*-test between two groups and 1-way ANOVA, followed by the Bonferroni’s *post-hoc* test among multiple groups by the Statview J-5.0 software (SAS Institute, Cary, NC, USA). A value of *P* < 0.05 was considered to be statistically significant. All values were expressed as the mean ± standard error of the mean (SEM).

## Results

### Assessment of lipid content and cell viability during the course of macrophage-derived foam cells formation and progress

**Fig. 1:**
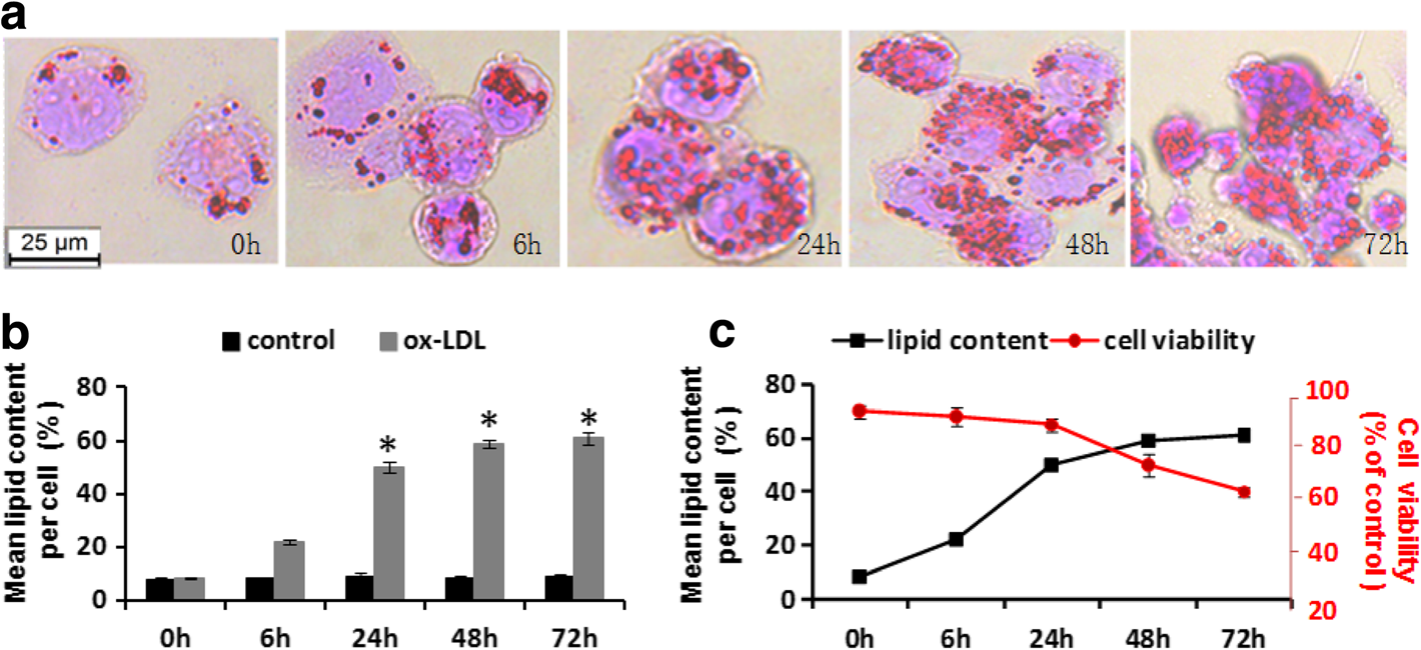
OxLDL-induced lipid accumulation and cell viability changes in human THP 1-derived macrophages. THP-1 macrophages were incubated with 80 ug/mL oxLDL for 0, 6, 24, 48, and 72 h. The intracellular lipid accumulation was determined by oil red staining and quantified with IPP software (**a**); (**b**) During the courses of foam cells formation and progress, cell viability was measured with MTT staining; (**c**) The data are expressed as means ± SD from at least 3 independent experiments. *P < 0.05 vs. 0 h. scale bars = 25 um

The qualitative observations via the Oil Red staining revealed the presence of a gradually increasing accumulation of larger-sized intracellular lipid droplets within the cytoplasm of the oxLDL-treated THP-1 macrophage (Fig. 1a). At the twenty-fourth hour, the mean lipid content per cell (52.0 ± 1.8 %) was significantly elevated compared to that of the control group (9.4 ± 0.9 %) (*P* < 0.05) (Fig. 1b), indicating that THP-1 macrophage derived foam cells had formed. However, after the 24 h, cell viability was trending down, which was an opposite tendency compared with that of intracellular lipid accumulation (Fig. 1c).

### Assessment of autophagy changing manner during macrophage-derived foam cell formation and progress

Autophagy is dynamic and encompasses autophagosome formation, cargo sequestration, and eventual lysosomal fusion/degradation occurring in rapid succession. To explore the involvement and manner of action of autophagy in the process of foam cell formation, we used Western blotting and mRFP-GFP-LC3 adenovirus infection to determine the amounts of autophagosomes and autophagic flux at different stages of foam cell progression, respectively.

**Fig. 2:**
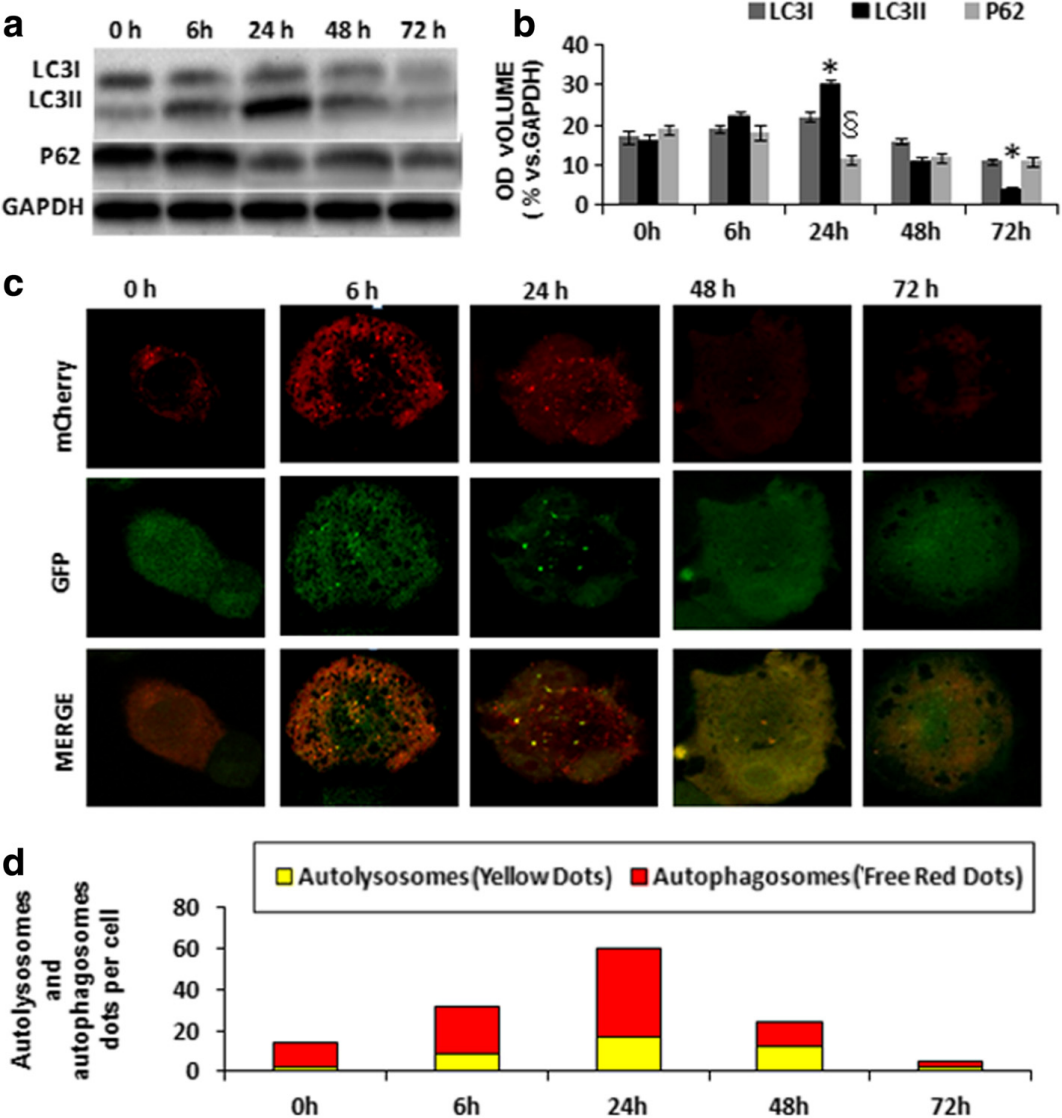
Assessment of autophagy in different stages of THP-M-derived foam cell formation. **a** Western blot analysis of p62 and LC3II/LC3I after THP-M incubation with oxLDL for 0 h, 6 h, 24 h, 48 h, and 72 h. The ratios of the mean grayscale of LC3I, LC3II, and P62 to GAPDH among groups are shown in (**b**); (**c**) Ad-LC3-GFP-mRFP infection measure autophagic flux intensity quantitative analysis; (**d**) the amounts of autolysosomes (*red*) and autophagosomes (*yellow*). The data are presented as mean ± SEM. ^*,§^ *P* < 0.05 vs control group

Western blot results demonstrated that the conversion of LC3-I to LC3-II in THP-M increased gradually within the first 24 h, which was consistent with the presence of defensive autophagy. Notably, P62 expression levels tended to attenuate with incubation time (Fig. 2a, b). However, after the treatment with oxLDL for 48 h, LC3-II levels significantly decreased from 30.2 ± 2.3 % (24 h) to 12.1 ± 1.5 % (48 h) (black column, *P* < 0.05), whereas there was no obvious change in the level of P62 in the high-lipid-accumulating foam cells compared with that at 24 h (light grey column, 13.2 ± 1.3 % vs 12.6 ± 1.2 %). Because GFP fluorescent protein is sensitive to acid, weakening of GFP signal may indicate that a lysosome has fused with an autophagosome to form an autolysosome, leading to GFP fluorescence quenching. As shown in Fig. 2c and d, in the first 24 h, the number of autolysosomes per cell increased from 3 dots (baseline) to 21 dots (the highest at the 24-h time point), and after 24 h, it decreased gradually to 4–5 dots per cell (at 72 h). These results showed that at the early stage, the amount of autophagosomes was increased, and autophagic flux intensity was accelerated, but at the mid-late stage of foam cell progress, autophagic flux was suppressed.

### Autophagy affects the fate of macrophage-derived foam cells

Many studies have provided evidence that autophagosomes exist in different stages of AS, indicating the involvement of autophagy in AS occurrence and development. However, the feasibility of autophagy as the target of treating AS is debated. One of the reasons may be that the effects of the same agent on different stages of foam cells formation are significantly different [17]. Therefore, according to the above results, we respectively selected early 6 and 48 h as regulatory “Key points” of foam cells life cycle to explore the effect of autophagy on the fate of THP-M-derived foam cells.

**Fig. 3:**
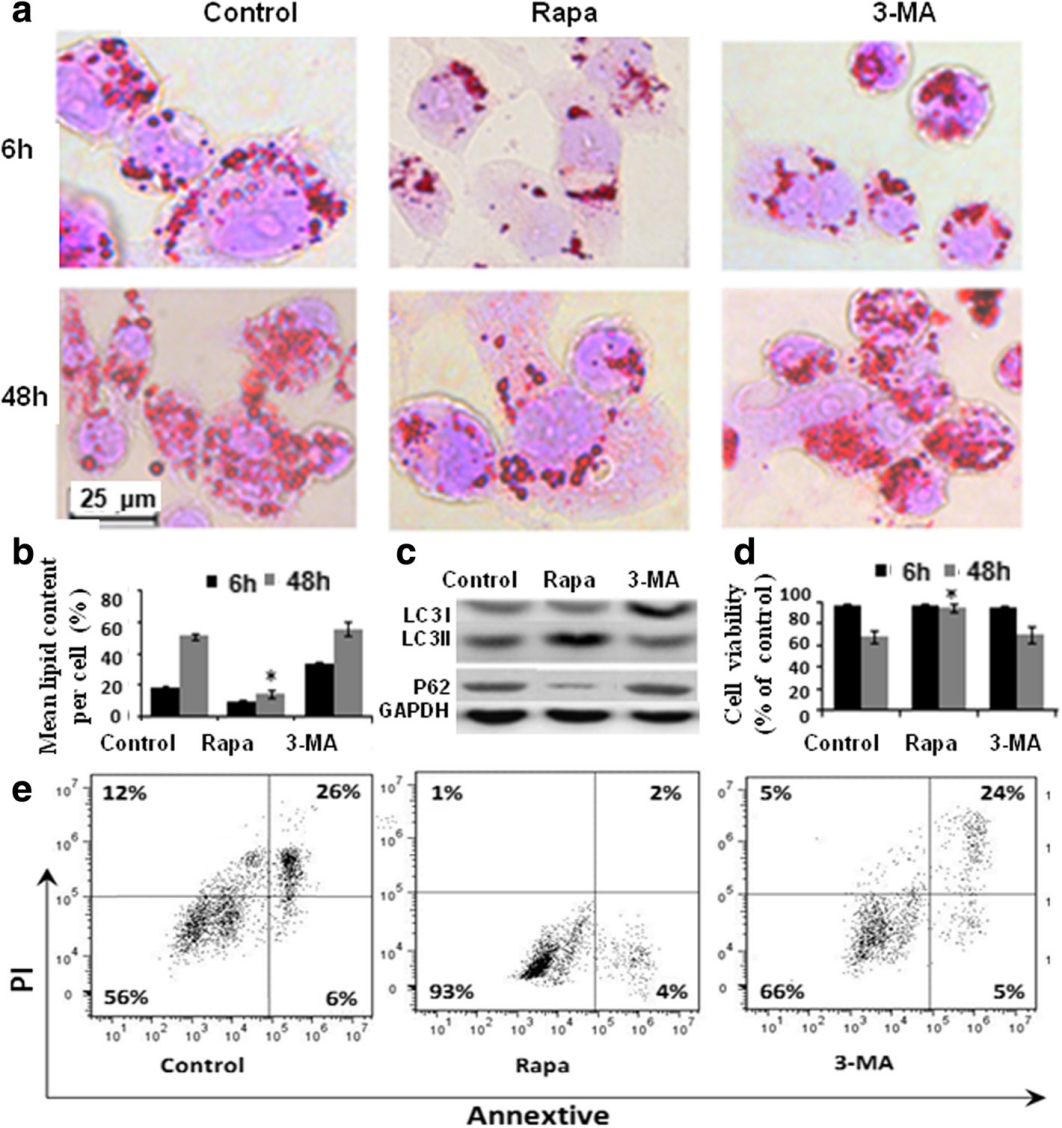
Effect of autophagy on the process of THP-M derived foam cells formation and progress. **a** Intracellular lipid content measurement by using oil red staining after incubation with oxLDL for 6 h (upper panel) or 48 h (downside panel) under conditions of autophagy activation (Rapa) or inhibition (3-MA); (**b**) Quantitative analysis of oil red positive area by using IPP software. The bars represent the mean of three independent experiments. * *P* < 0.05 vs. control group; (**c**) Western blot analysis for the regulatory effect of Rapa or 3-MA on autophagy specific molecules; (**d**) The cell viability was detected by an MTT assay. The results showed that the Rapa treatment improved the viability of mid-late stages foam cells in the 48-h group, but exerted no effect on the foam cells that were cocultured with oxLDL for 6 h; (**e**) The AV-PI two-color dot plot for cell viability to further test the effect of rapa on mid-late staged foam cells. The values are the means ± SD of 3 separate experiments; *P < 0.05 vs. control group

During the process of foam cell formation, co-culturing THP-M with oxLDL for 6 h in the presence of autophagy activator rapamycin (80 ug/mL) markedly decreased intracellular lipid content, while the autophagy inhibitor 3-MA (10 mM) considerably increased the intracellular lipid content (upper panel in Fig. 3a, b). Furthermore, Western blot results demonstrated that 80 μg/mL of rapamycin successfully upregulated the conversion of LC3I to LC3II, while P62 levels in the cytoplasm decreased significantly compared with that of the control. The application of 3-MA at 10 mM effectively inhibited autophagic process (Fig. 3c), which was consistent with previous studies [18, 19].

During the process of foam cell progress, THP-M was co-cultured with oxLDL for 48 h. As shown in Fig. 3, Rapa not only reduced significantly the lipid content in the cytoplasm (downside panel in Fig. 3a, b), but also increased the viability of mid-late-THP-M derived foam cells (Fig. 3d, e). To determine if the inhibition of macrophage autophagy affected the fate of mid-late THP-M-derived foam cells, we treated THP-M with autophagy inhibitor 3-MA. Not only was the lipid content increased, but also cell viability was significantly impaired, suggesting the fate of THP-M-derived foam cells was associated with autophagy.

### Autophagy influenced atherosclerotic lesion area and stability

**Fig. 4:**
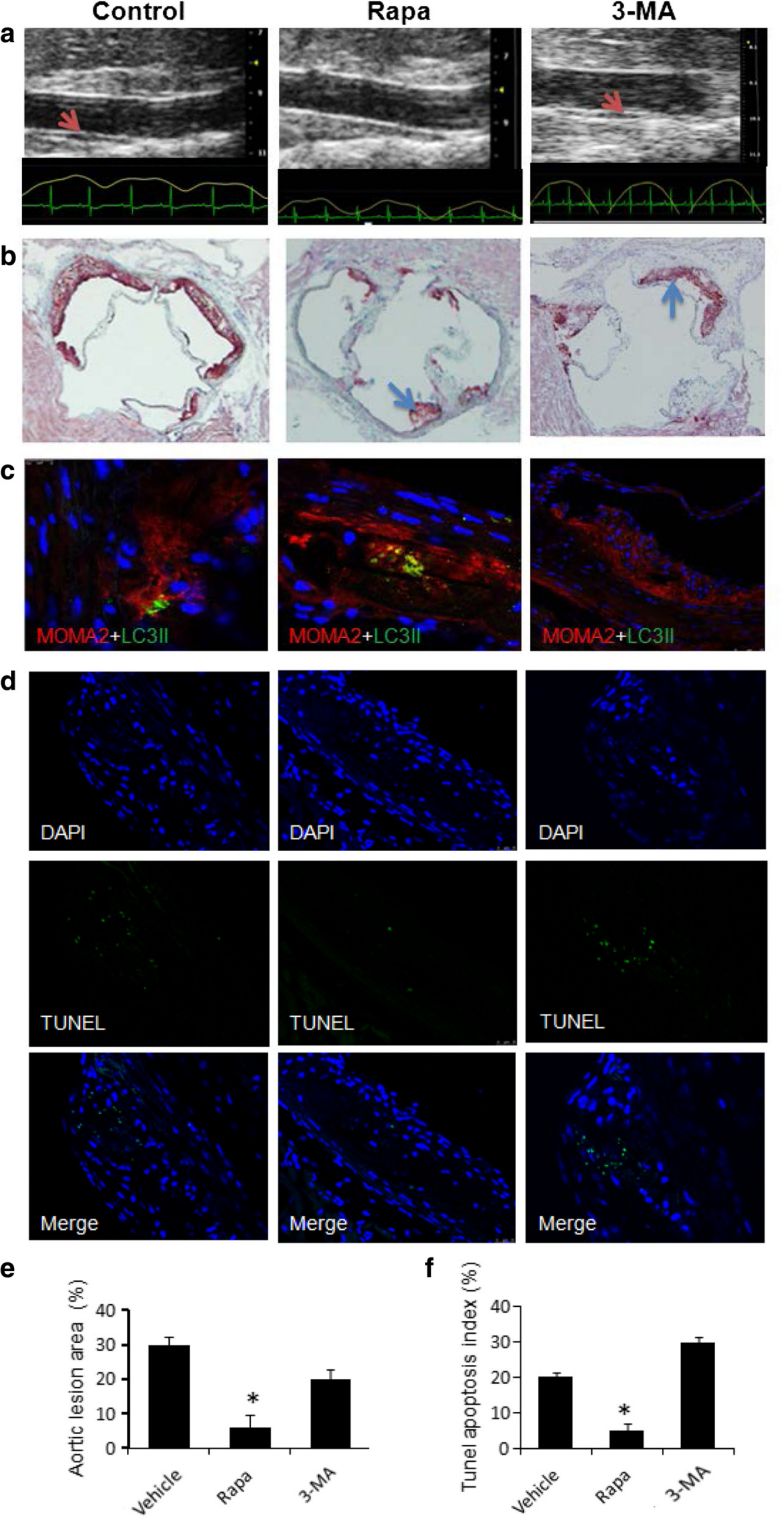
Autophagy influences atherosclerogenesis. **a** Atherosclerotic lesions (*red arrows*) in the aorta were examined after 8 weeks of treatment by an intravascular ultrasonic imaging system; (**b**) Atherosclerotic lesion formation (*blue arrows*) under Oil Red O staining in ApoE−/− mice at 16 weeks after administration; (**c**) Representative images and morphometric analysis for the macrophages specific marker MOMA2 (*red*) and autophagy specific marker LC3II (*green*) staining from the aortic root; (**d**) TUNEL assay and DAPI staining. Green spots represent apoptotic bodies and blue spots represent cell nuclei; (**e**) Statistical data of atherosclerotic lesion area in the aortic root; (**f**) Statistical data of TUNEL positive cells. Apoptosis rate was calculated based on the results of the TUNEL assay. The pictures are representative of multiple sections of aortas from different groups of mice (at least 5 in each group);*P < 0.05 when compared with Vehicle group; scale bar = 80 um. Values are the mean ± SD of 3–5 separate experiments; *P < 0.05 vs. control group

Atherosclerotic lesions in the apoE−/− mice aorta were examined by an intravascular ultrasonic imaging system eight weeks following treatment with rapa, 3-MA or 0.9 % normal saline (Vehicle control). The results showed that the atherosclerotic lesions in the abdominal aorta of the control group were much larger than those of the Rapa group, but were significantly smaller than those in the 3-MA treated group (Fig. 4a). After 16 weeks of the treatment, mice were euthanized. The atherosclerotic lesions were assayed by aortic root Oil Red O staining techniques (Fig.4b). These observations are supported by a recent report demonstrating a role for autophagy in foam cell formation [20]. The atherosclerotic lesion area was evaluated as a ratio to the area of the whole aorta. The aortic root lesion area was reduced significantly by rapamycin (*P* < 0.05). There was a statistically significant difference between Rapa and 3-MA groups concerning the atherosclerotic lesion area. Our findings also supported the notion that autophagy was substantially impaired in the advanced stages of atherosclerosis, and autophagy deficiency promoted atherosclerosis.

To determine which cells in atherosclerotic plaques express autophagy markers, we characterized ApoE−/− atherosclerotic aortic roots by immunofluorescence. The autophagy markers LC3 (Fig. 4c) were concurrently imaged with MOMA-2 (an antibody that recognizes monocyte-macrophages). These results indicated that monocytes/macrophages were the predominant cell type expressing autophagy markers in the plaque and were likely responsible for progressive autophagy deficiency. The TUNEL assay (Fig. 4d) showed that the apoptotic cell number in the rapa-treated group was considerably lower than that in the control group, indicating that Rapa can prevent mid-staged AS from plaque progression and enhance the stability of plaques.

### Potential mediators of autophagy biological effect

**Fig. 5:**
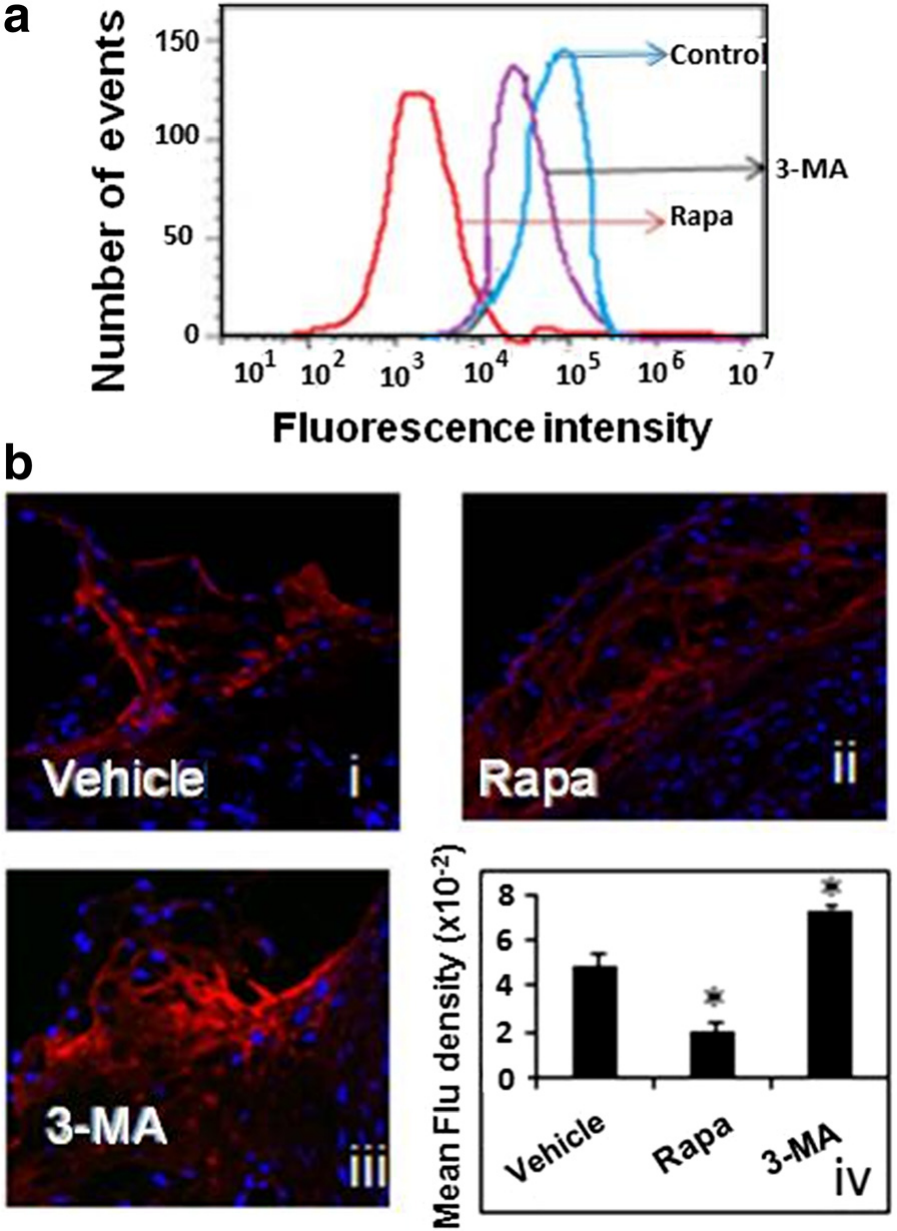
Reactive Oxygen Species is a potential mediator for the protective effect of autophagy. **a** Measurement of the ROS production in THP-1 macrophage foam cells of different groups using flow cytometry; (**b**) Confocal reflectance microscopy of formaldehyde-fixed frozen-section aortic roots from Western diet-fed control, rapa, and 3-MA treated ApoE−/− mice. Reflectance images for a representative area of the aortic root are shown in B-i, −ii, and −iii, and quantified by using the IPP image analysis software. Values are given as mean ± standard deviation of the mean (*n* = 3). *P < 0.05 versus the control

One of the proposed mechanisms for foam cell apoptosis has been the presence of reactive oxygen species (ROS) [21, 22]. Thus, we looked for signatures of ROS activation in the THP-M-derived foam cells and atherosclerotic plaques. To understand the mechanistic basis of autophagy in the progress of atherosclerosis, we examined intracellular ROS production under upregulated autophagy (Rapa-treated group) or downregulated autophagy conditions (3-MA-treated group). As depicted in Fig. 5a, the ROS level in the Rapa group was suppressed to 54 %. There was a significant increase of the mitochondria-derived superoxide anion levels in 3-MA groups (*P* < 0.05), which was consistent with that in the autophagy specific silencer (Atg 5-siRNA) treated group (Additional file 1: Figure S1). All of the above data indicated that maintenance of autophagy activity could largely prevent mitochondria-derived superoxide production. Dihydroethidium (DHE) staining was used to detect ROS production of atherosclerotic aortic roots since DHE can react with ROS and form ETH that binds to DNA and produces a red fluorescence signal. The mean fluorescence density in the Rapa-treated group was significantly lower than that in the vehicle control group, but there was a marked rise in the amount of 3-MA-treated mouse plaques (Fig. 5b).

## Discussion

In the present study, for the first time, we clarified the changing pattern and function of autophagy in the development and progress of macrophage-derived foam cells and validated the hypothesis that in middle-late foam cells, autophagy was restrained, intracellular accumulation of lipid droplets and mitochondria clearance impaired, and a large number of reactive oxygen species generated. Cell viability decreased, which may be the most important factor that contributed to plaque progress and instability, autophagy may be a potential target for AS treatment. Therefore, our study has discovered a novel perspective to understand the mechanism of plaque stability and provides a clue for developing comprehensive methods for diagnosis and treatment of AS.

Autophagy is dynamic and has also been shown to be directly involved in lipid homeostasis [23, 24]. This type of autophagy, called lipophagy, was first found in the liver and has now become a subject of intense research interest with potentially profound implications for the treatment of the diseases associated with dyslipidemias, such as diabetes and atherosclerosis [25, 26]. In this examination, to clarify the changing pattern and function of autophagy in AS, THP-1 macrophage was induced with ox-LDL (80 μg/mL) into different stages of foam cell models. When cocultured with ox-LDL for 6–72 h, THP-M cell morphology and lipid content were in line with the characteristics of foam cells at the early stage, middle stage, or late stage. After 48 h, irreversible death occurred in a large number of foam cells. Therefore, in this study, 6 or 48 h were selected as optimal time points to regulate autophagy and investigate its effect.

It is well known that the autophagy process includes autophagosome formation, cargo sequestration, and eventual lysosomal fusion/degradation occurring in rapid succession. Autophagosome-lysosomal fusion is the essential step. LC3-II is a relatively sensitive biochemical marker of autophagy. Ad-LC3-GFP-mRFP is a reporter system. LC3 is distributed in the cytoplasm under normal conditions (LC3-I), but when autophagy is induced, LC3-I is modified to become LC3-II, which is integrated into the autophagosome membrane [26]. In the early-staged foam cells, with prolonged oxLDL incubation time, autophagosomes continued to increase, and the autophagic flux intensity was gradually enhanced, characterized by an increased proportion of LC3II/I and percentage of mRFP-positive cells. When THP- M cells were incubated with oxLDL and Rapa for 6 h, the oil red staining assay results showed that the lipid deposition in cells was limited, and while intracellular autophagosome and autophagy-lysosome quantity increased gradually, the cell membrane remained intact. When oxLDL and THP-M interaction lasted beyond 6 h, autophagy was restrained; the deposition of lipid droplets in cells increased obviously, and the rapa treatment at this stage not only corrected the dysfunctional autophagy and decreased the formation rate of foam cells.

Komatsu et al. found that homeostatic levels of p62 control cytoplasmic inclusion body formation in autophagy deficient mice. p62, a chaperone that shuttles intracellular protein aggregates into autophagosomes for degradation, has emerged as a useful marker of autophagic status [27–30]. Since the entire p62/SQSTM1-protein aggregate complex is degraded after engulfment by the autolysosome, p62 level is inversely correlated with autophagic flux, i.e., the increases of p62 level indicate defective autophagy [29, 30]. Our results also showed that P62 level decreased at the early stage of foam cell formation. Published evidence supports this possibility. The cells in atherosclerotic plaques develop progressive lysosomal dysfunction with features resembling a lysosomal storage disease [31]. The accumulation of unhydrolyzed cholesteryl esters and trapped/poorly translocated free cholesterol in lysosomes has been described in macrophage foam cells [32–37]. These unmetabolized substituents may impair the lysosomal degradation capacity of lipases and other enzymes, including proteases. Our observation that p62 accumulates in the advanced atherosclerotic plaques is consistent with this concept and verify that autophagy is inhibited in advanced plaques.

The general consensus on the function of autophagy is that in contrast to basal autophagy, the excessive stimulation of autophagy may cause autophagic cell death. This is logical since both autophagy and cell death may be activated in response to similar stress conditions [38, 39]. Therefore, the proper regulation of autophagy at the right time point is important. Facing the inhibited autophagy in advanced atherosclerotic plaque, an important question exists whether it is feasible to consider autophagy as an intervention target?

In this study, we fed Apo E −/− mice (known to be susceptible to atherosclerosis) with a Western diet for 16 weeks (middle phase of spontaneous AS) and treated them with an autophagy stimulator (Rapa) or autophagy inhibitor (3-MA) for 8 weeks. Then, assessments were performed of plaque area/stability, the types of inflammatory cells infiltration, ROS levels, and cell viability in the aortic roots of mice in the different groups.

According to our results, upregulation of autophagy to its normal level did decrease the apoptosis rate in mid-late phase THP-M foam cells and suppress the atheroma progression. However, what is the nature of this action? We found that the autophagic removal of damaged mitochondria (also called mitophagy) was enhanced, and, in turn, reduced reactive oxygen species (ROS) production. It has been demonstrated that release of ROS and DNA from damaged mitochondria can activate inflammasomes, a process that contribute to plaque formation and progression [22, 40]. Therefore, the possible mechanism of the protective effect of rapa is through upregulation of autophagy and decrease of mitochondrial ROS. On the other hand, the observation that plaques treated with the autophagy inhibitor 3-MA increased the levels of markers of protein oxidation and superoxide production lends credence to this possibility.

There were several limitations of this study. This work was focused on studying the function of autophagy on the fate of macrophage-derived foam cells and exploration of the most relevant intervention time. Although we have used the respective autophagy agonists and inhibitors to observe the effect of autophagy on the foam cell development in Ox-LDL-treated THP-1 macrophages or the progress of atherosclerotic plaques in high fat diet Apo E −/− mice, we did not use foam cells derived from vascular smooth muscle cells, which are also important for atherosclerosis plaque formation and maintenance of the stability of atherosclerotic lesions. In addition, if macrophage-specific autophagy defects in mice can be used to repeat and further develop the preliminary findings of this study in the future, the results would be more persuasive and convincing.

## Conclusions

The fate of macrophage FCs was associated with autophagy. Autophagy might be a promising intervention target for AS treatment.

## Abbreviations

DHE, dihydroethidium; FC, foam cell; LC3, microtubule-associated protein light chain 3; oxLDL, oxidized low-density lipoprotein LDL; PMA, phorbol 12-myristate 13-acetate; ROS, reactive oxygen species

## Additional file

Additional file 1: Figure S1. Atg 5-siRNA had an enhancing effect on the production of mtROS similar to that of 3-MA. (A) Measurement of the mtROS production in THP-1 macrophage foam cells of different groups using flow cytometry. (B) Confirming the influence of Atg 5-siRNA on the expression of Atg 5 using Western blotting. Scambled siRNA is the negative control siRNA with the same nucleotide composition as Atg5 siRNA but which lacks significant sequence homology with the genome. (TIF 874 kb)
