# Supplementary figures and images for: Autophagy is associated with cell fate in the process of macrophage-derived foam cells formation and progress
Source: J Biomed Sci. 2016 Jul 30;23:57. doi: 10.1186/s12929-016-0274-z (PMC4967324; doi:10.1186/s12929-016-0274-z)

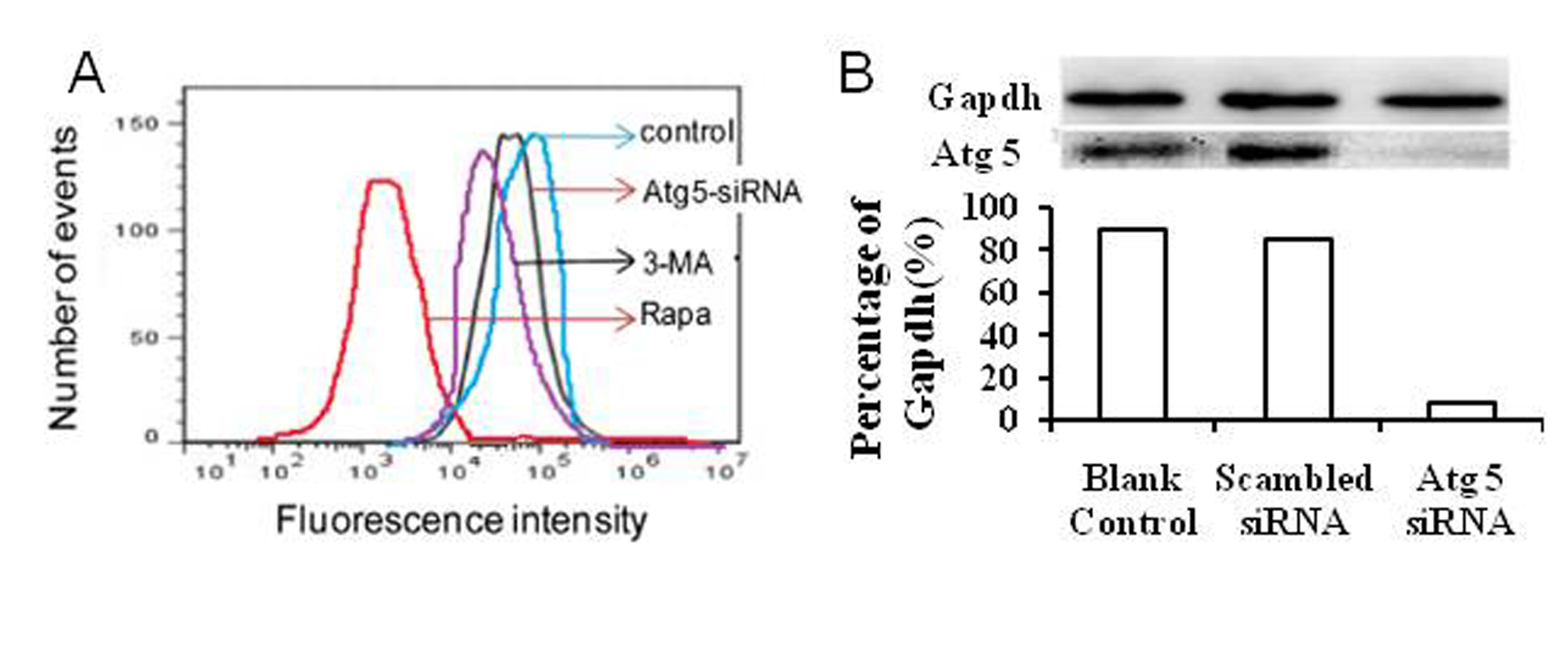

Supplement: Additional file 1: Figure S1. — Atg 5-siRNA had an enhancing effect on the production of mtROS similar to that of 3-MA. (A) Measurement of the mtROS production in THP-1 macrophage foam cells of different groups using flow cytometry. (B) Confirming the influence of Atg 5-siRNA on the expression of Atg 5 using Western blotting. Scambled siRNA is the negative control siRNA with the same nucleotide composition as Atg5 siRNA but which lacks significant sequence homology with the genome. (TIF 874 kb) [file 12929_2016_274_MOESM1_ESM.tif]
